# Wildfires as a Source of Potentially Toxic Elements (PTEs) in Soil: A Case Study from Campania Region (Italy)

**DOI:** 10.3390/ijerph20054513

**Published:** 2023-03-03

**Authors:** Lucia Rita Pacifico, Antonio Pizzolante, Annalise Guarino, Antonio Iannone, Mauro Esposito, Stefano Albanese

**Affiliations:** 1 Department of Earth, Environmental and Resources Sciences, University of Naples Federico II, 80126 Naples, Italy; 2 Istituto Zooprofilattico Sperimentale del Mezzogiorno, 80055 Portici, Naples, Italy; 3 Centro di Referenza Nazionale per l’Analisi e Studio di Correlazione tra Ambiente, Animale e Uomo, IZS Mezzogiorno, Via Salute 2, 80055 Portici, Naples, Italy

**Keywords:** risk assessment, environment, wildfires, enrichment factor, potentially toxic elements, principal component analysis, geochemical mapping, mercury

## Abstract

The worldwide increase in fire events has attracted global attention, as potentially toxic elements (PTEs) have been widely recognised within the produced ash. Ash is transported, dispersed by wind, and deposited into the soil and surficial waters even far from fires. Considering that their composition can be enriched in PTEs, they represent a potential hazard for humans and other animals exposed to airborne particles and, afterwards, to resuspended matter, even at a considerable distance from the source. This study aimed to assess the environmental impact of fire events that occurred during the 2017 summer season at two different sites in the Campania region (Southern Italy). One of the fires affected a waste disposal site west of Caserta, and one involved a forest on the slopes of Mt. Somma-Vesuvius, a few kilometres southeast of Naples, the regional capital. Changes to the PTE concentration in the topsoil in the surroundings of both sites, after the fire events, were investigated. Enrichment factors (EFs) of a selection of PTEs were determined by comparing geochemical data from two sampling campaigns, one completed before and one after the fire events. A combined application of multivariate statistics (based on robust principal component analysis; RPCA) and geospatial analysis was used to determine the materials affected by the fire on the slopes of Mt. Somma-Vesuvius, and roughly locate their place. Specifically, a statistically significant enrichment of Hg was identified in the topsoil of both study areas. In addition, in soil samples collected at Mt. Somma-Vesuvius, more PTEs showed significant changes in their concentration. For both areas, Hg enrichments were related to the deposition of ash proceeding from waste burning; furthermore, as regards the soil of the Vesuvian area, Cr and Cd enrichments were associated with the fallout of ash generated during biomass combustion, and the increase in Cu and Zn concentrations was linked to the burning of crops on cultivated lands. Apart from the specific results obtained, concerning the examined case studies, the methods applied can be seen as a reliable option to determine the compositional characteristics of materials burned during a fire event, even with the prospect of improving the eventual assessment process of the related environmental hazards.

## 1. Introduction

Climate change and intentional illegal fires are increasing the number of wildfires worldwide, and, unfortunately, this amount is bound to rise in the following years [1]. Reduced rainfall, higher temperatures, and windy conditions, especially during the hot season, increase the overall chance of both fire ignition and diffuse outbreaks. In the last decade, the fire season included a string of abnormally large fires across the globe: 2019 (Alaska, Russia), 2018 (Fennoscandia, Russia), 2017 (Greenland), 2016 (Canada), 2015 (Alaska), and 2014 (Canada) [2]. In 2019 (from July to September), an extreme biomass burning event occurred in the Amazonian rainforest, resulting in the largest fire in the area since 2013, with cities darkened by smoke even thousands of kilometres from the source. In Australia, the “black summer fire season” (2019–2020), which involved extensive areas on the eastern seaboard as well as some territories in the south and the middle-east [3,4,5], is widely considered one of the worst that occurred in recent memory.

Even in the Artic regions, wildfires have increased in recent years, due to higher temperatures, probably linked to global warming. For example, in 2020, a fire event in Siberia, in the northern-central Russian Arctic, burned tens of millions of hectares of forest [6].

Fire events represent a problem of global interest, since during their occurrence, several chemicals, such as potentially toxic elements (PTEs), are released into the environment. 

Potentially toxic elements (PTEs), which are both metals and metalloids, include arsenic (As), antimony (Sb), cadmium (Cd), mercury (Hg), lead (Pb), chromium (Cr), copper (Cu), nickel (Ni), selenium (Se), thallium (Tl), tin (Sn), vanadium (V), and zinc (Zn). These elements may harm humans and animals when their absorbed dose exceeds the relative reference values [7,8]. In general, exposure to excessive amounts of PTEs may cause, depending on the element, respiratory, endocrinal, and genetic disorders, cancer, seizures, hypotension, anaemia, osteomalacia, skin and hair problems, cardiac arrhythmias, and diseases related to the stomach, kidneys, reproductive organs, and nervous system [9] (see more details in Table S1).

Ash can release PTEs into the soil, sediments, or surficial waters, even after long-range airborne transport.

For example, in 2013, in a study conducted in the West Attica region, chemical evidence was found that the ash generated by wildfires was carried by the wind far away from the source and deposited in neighbouring urban areas, not directly involved in the event. The deposition of ash was detected since soils were locally enriched by some PTEs related to the nature of burned materials [10].

The chemical composition of ash may depend on several variables, including the intensity of combustion, the composition of the soil at the fire location and, most importantly, the composition of the burned material [11]. In the case of biomass, the concentration of some PTEs seems to change according to the type of wood in combustion [12,13]. Even for waste, the presence of specific elements in the ash may depend on the material’s nature (e.g., general solid putrescible materials, electronic devices, special or hazardous waste) [14].

Furthermore, the transfer of elements from ash to soil can also modify the soil’s chemical and physical characteristics, influencing the development and growth of local microorganisms and vegetation [15].

**Figure 1:**
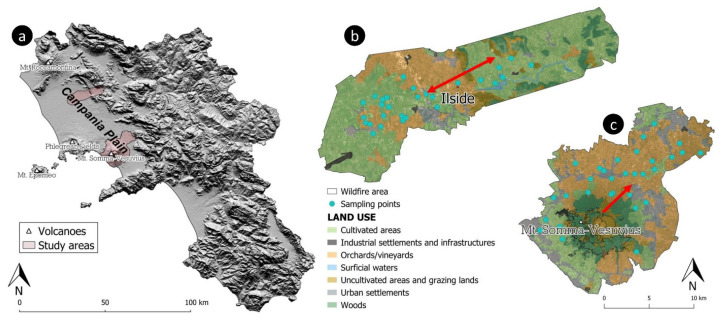
(**a**) DTM of Campania region, with the indication of the study areas; (**b**) soil sampling map at the Ilside site; (**c**) soil sampling map of Mt. Somma-Vesuvius slopes. Red arrows on maps represent the main wind directions during the fire events.

During the 2017 summer season, the Campania region (in southern Italy) was involved in two severe wildfires (Figure 1): one occurred on the slopes of the Mt. Somma-Vesuvius volcano (for seven days, between 11 and 17 July), one affected an industrial waste disposal site in the Caserta province (on 11 July).

This work aimed to verify if any relevant compositional change occurred to soil affected by ash fallout in the surroundings of the fire locations, by comparing PTE concentrations in the topsoil before and after the events. 

Enrichment factors of PTEs in soil were determined, and multivariate analysis was applied to eventually identify any elemental association which could support the discrimination of the different materials involved in the fires.

## 2. Materials and Methods

### 2.1. Study Areas

Campania is one of the most populated regions in Italy. It is characterised by a Mediterranean climate, with hot, dry summers and moderately cool, rainy winters. The study areas are both located within a flat and wide coastal belt (Campanian Plain) that goes from the Volturno River plain (in the north) to the Sarno River basin (in the south) (Figure 1a). The Campanian Plain is a tectonic depression characterised by a general tensional tectonic regime, with NE–SW and NW–SE regional fault systems, which produced a graben affecting the carbonate basement. Two Quaternary volcanic complexes are in the plain: the Mt. Somma-Vesuvius and the Phlegrean Fields volcanic district, including Ischia island. The pyroclastic fall deposits, which represent one of the main products of volcanic activity, together with the potassic and ultrapotassic lavas, are the outcropping geological material that features on the plain [16]. The soil of the plain is generally developed on pyroclastic materials and retains their geochemical characteristics, being characterised by andic properties [17].

Densely populated urban settlements and industrial sites are located within the plain; they are usually adjacent to territories extensively dedicated to agriculture, representing a significant resource for the local economy [18].

#### 2.1.1. Ilside Site

The Ilside site was an industrial waste storage and disposal site managed by an international company, located in Bellona (Caserta province), in the NE sector of the Campania Plain (Figure 1a): the Volturno River is immediately north of the site, while the Trebulani Mountains are to the east. 

Ilside was active from 2000 until 2012 and, after its decommissioning, until 2017, different types of waste were illegally disposed into the area, as reported by local newspapers. According to the regional rural territorial system [19], the site is included in the Domitio Littoral and Volturno Plain (Figure 1b); the Ilside plant was also included in the Domitio–Flegreo Littoral Site of National Interest (SIN), which the Italian Government has outlined due to its high contamination potential (L. 426/1998). Across the area, illegal waste disposal and uncontrolled burning of agricultural and industrial wastes historically affected the overall environmental equilibrium.

On 11 July 2017, a *localised fire* occurred at the Ilside plant. The ash cloud generated by the event invaded the neighbouring municipalities, brought by the wind blowing towards the SW and NE. After the fire event, the Environmental Protection Agency of Campania Region (ARPAC) reported that 4500 tonnes of waste had been burned. Of the total amount of the burned material, 1500 tonnes were made of urban waste and hazardous and non-hazardous special waste, while the rest (3000 tonnes) was previously burned materials mixed with extinguishing powders.

#### 2.1.2. Mt. Somma-Vesuvius Slopes

Mt. Somma-Vesuvius is a stratovolcano that has been quiescent since the last eruption occurred in 1944. Together with the Phlegrean Fields, Procida, and Ischia, it is one of the four volcanoes within the Neapolitan area. It is located about 20 km from Naples and covers an area of almost 150 km^2^, reaching a maximum height of 1281 m a.s.l. The volcanic complex includes Mt. Somma, the older volcanic edifice, with a summit caldera formed during several eruptive phases, and the recent cone of Vesuvius, that grew up within the caldera after the 79 AD Pompeii eruption [20].

The volcano is the primary naturalistic resource of the namesake national park (Figure 1c). The park hosts diversified natural and geologic landscapes and covers an area of 3800 ha; its vegetation is dominated by pure and mixed broadleaved, even-aged monospecific, mixed coniferous stands and scrubland. Specifically, due to the different exposure of the slopes, the volcanic complex can be divided into two main sectors: a sunnier and arid one, covered by the typical spontaneous Mediterranean vegetation, pinewoods, and holm oak forests; and a more humid one, with woody vegetation, mixed woods of chestnut, oak, alder, maple, and holm oak trees.

During the week between 11 and 17 July 2017, the slopes of Mt. Somma-Vesuvius were traversed by a *widespread wildfire*. The fire started on the SE sector of the volcanic complex and caused extensive damage to the forest heritage. The ash resulting from the burning of the Mediterranean scrub were dispersed into the atmosphere, generating a notable plume that extended towards the NE, where several human settlements are located.

### 2.2. Sampling and Analysis

In 2017, the Experimental Zooprophylactic Institute of Southern Italy (IZSM) carried out two soil sampling activities in the areas adjacent to both fire zones. The first soil sample collection was carried out before the fire events, as a part of the more general regional survey completed in the framework of the Campania Trasparente project [21]; sample collection after the fires was, by contrast, planned, and completed with the specific aim of carrying out the present study. For both areas, samples collected after the fires, were taken in the exact locations as those sampled during the first survey. Sampling sites were selected paying attention to collecting the soil along the trajectories followed by the smoke plumes (Figure 1b,c). Regarding the Ilside site, the main wind direction on 11 July 2017 was SW to NE for most of the day and NE to SW at night. For the Vesuvian area, during the seven days of wildfires, the pre-eminent wind direction was SW to NE [22,23].

One hundred and twenty topsoil samples were collected at the two sites (Figure 1b,c), within a depth ranging from 0.10 to 0.15 m below ground level. A composite sample of about 1.5 kg was collected at each location. A unique alphanumerical code identified every sample, and additional information was collected, including site geographical coordinates and physical, geological, and pedological characteristics of the surrounding areas.

An aliquot of each sample, of about 30 g, was prepared and sent to the Bureau Veritas Analytical Laboratories Ltd. (Vancouver, BC, Canada) for analytical determination. Samples were digested through an aqua regia solution, followed by inductively coupled plasma mass spectrometry (ICP-MS) and inductively coupled plasma emission spectrometry (ICP-ES). A total of 46 elements were determined (Ag, Al, As, Au, B, Ba, Be, Bi, Ca, Cd, Ce, Co, Cr, Cs, Cu, Fe, Ga, Hf, Hg, K, La, Li, Mg, Mn, Mo, Na, Nb, Ni, P, Pb, Rb, S, Sb, Sc, Sn, Sr, Te, Th, Ti, Tl, U, V, W, Y, Zn, and Zr).

For this study, only the concentrations of 15 elements (i.e., As, Be, Cd, Co, Cr, Cu, Hg, Mo, Ni, Pb, Sb, Sn, Tl, V, and Zn), considered as PTEs by Italian law, were used.

### 2.3. Statistical Analysis and Geochemical Mapping

Univariate statistical analysis was performed on datasets of both study areas, and several position and morphological indices were determined, to have a general view of their distribution. For both study areas, using the R software, a non-parametric Wilcoxon test was performed on matched pair groups referring to single elements, to assess if ash deposition brought sensitive changes to their concentrations in soil (Table 1).

The Wilcoxon test is used to compare the locations of two populations, two related samples, or matched samples and to determine if the datasets are different from each other in a statistically significant manner. The test produces a statistic value (i.e., z-score) which is converted into the probability (i.e., *p*-value) that a *null* hypothesis is true. In our case, the *null* hypothesis is that the considered data populations have no statistical differences. A *p*-value ≤ 0.05 (significance level α) expresses significant differences among the examined populations. The Wilcoxon test is generally preferred when the data do not follow a normal or lognormal distribution [24]. 

The statistical distribution of the pair data was also evaluated, utilising both density curves and boxplots to detect possible outliers and extreme values. Comparing PTEs compositions in the pre- and post-event datasets, enrichment factors (EFs) were calculated at each sample location.

Specifically, the EF was estimated through the following equation (Equation (1)):

(1) $$\mathrm{EF}=\frac{{\mathrm{CPTE}}_{before}/{\mathrm{CREF}}_{before}}{{\mathrm{CPTE}}_{after}/{\mathrm{CREF}}_{after}}$$

where:

- CPTE*_before_* and CPTE*_after_* are, respectively, the concentration values of the PTE before and after the fire;
- CREF*_before_* and CREF*_after_* are the values of a normalising element taken as reference.

The normalising element was selected as that with the lowest coefficient of variation (CV) [25] (Table 1) among all the geochemical variables in the dataset.

A robust principal component analysis (RPCA) was also performed on the EF values of the Vesuvian area, to better understand the correlation structure of the data through a reduced number of linearly uncorrelated variables (i.e., principal components; PCs), limiting the loss of information as much as possible.

The RPCA method was chosen since it can generate PCs poorly affected by the presence of outliers in the datasets, as was the case. The RPCA was performed in line with the method suggested by Hubert et al. [26], through the “pcahubert” function, available in the “RRCOV” package within the R software framework. This method computes the variable (vector) loadings using projection pursuit technique and the minimum covariance determinant (MCD).

A biplot was generated, to integrate both the loading of the variables’ (EFs) and the observations’ (samples) scores concerning the most relevant PCs found by the RPCA. The scores of single samples, relative to PC1, PC2, and PC3, were also extracted, and spatially interpolated via the inverse distance weighting (IDW) method in QGIS 3.22. Intervals on the interpolated grid were assigned based on a symmetrical principle, considering that the contribution of a sample becomes irrelevant when the sample score is close to zero.

## 3. Results 

### 3.1. Ilside Site Surroundings

**Figure 2:**
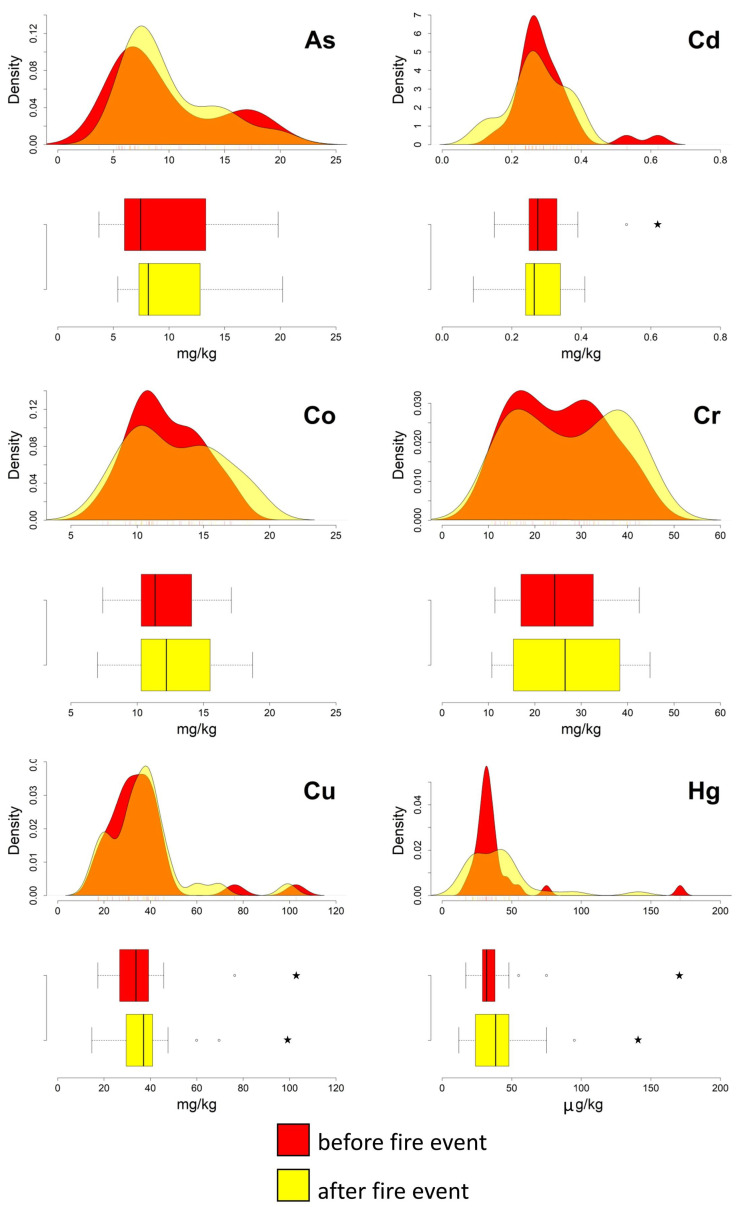
Ilside site. Boxplots and density curves of selected PTEs (As, Cd, Co, Cr, Cu, and Hg) in the soil before and after the fire event. Circles and pentagrams represent outliers and extreme values, respectively.

**Figure 3:**
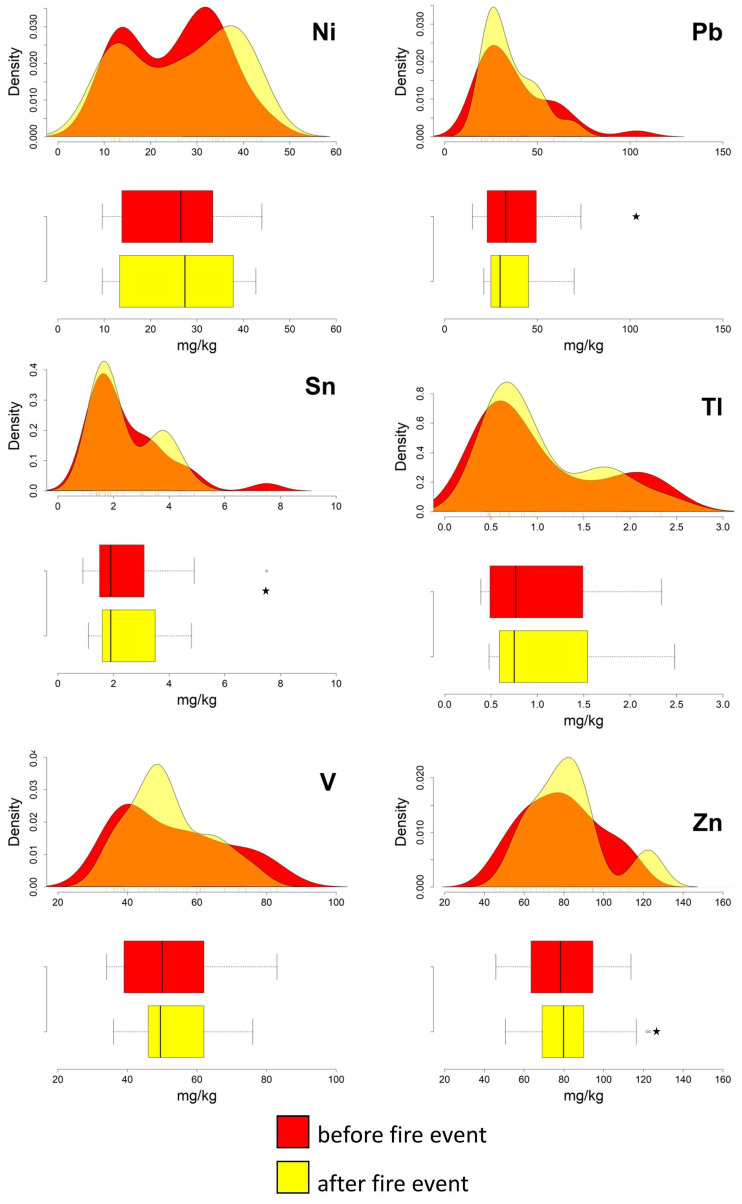
Ilside site. Boxplots and density curves of selected PTEs (Ni, Pb, Sn, Tl, V, and Zn) in the soil before and after the fire event. Circles and pentagrams represent outliers and extreme values, respectively.

The boxplots presented in Figure 2 and Figure 3 show that, after the fire event, Cd, Co, Cr, Cu, Hg, Ni, Sn, and Tl are characterised, at different degrees of significance, by a widening of the interquartile ranges. This latter condition is primarily due to a shift toward higher values of the third quartile (75th percentile). In addition, compared to the data from the first sampling campaign, in the post-fire data the medians of Co, Cr, Cu, Hg, Ni, As, and Zn tend to have a higher value (Table 1), and the standard deviation of Hg is doubled.

The pre- and post-event density curves show relevant changes for Hg, Sn, and Zn; the latter two elements even pass from a unimodal to a bimodal distribution. Slight variations are shown by the remaining PTEs (Figure 2 and Figure 3).

Some outliers were also detected in both pre- and post-event datasets, for Cd, Pb, Sn, and Zn, suggesting a pre-existing condition of diffuse contamination, probably associated with the proximity of the sampling points to highly urbanised areas [27,28].

The results of the Wilcoxon test (Table 1) support the evidence that the Hg data are characterised by pre-event and post-event distributions differing statistically significantly. For the remaining elements (i.e., As, Be, Cd, Co, Cr, Cu, Mo, Ni, Pb, Sb, Sn, Tl, V, and Zn), although some changes were detected, the existence of significant variations in their soil abundance following the deposition of the ash, cannot be unequivocally established.

Enrichment factors (EFs) were calculated for all the PTEs (Table S2). Iron was chosen as the normalising element, due to its low CV value in the pre- (6.44%) and the post-fire (7.12%) event datasets. Mercury EFs range from 0.35 to 3.49, and the mean (1.09) and maximum (3.49) suggest an element accumulation in the topsoil surrounding the study area.

**Figure 4:**
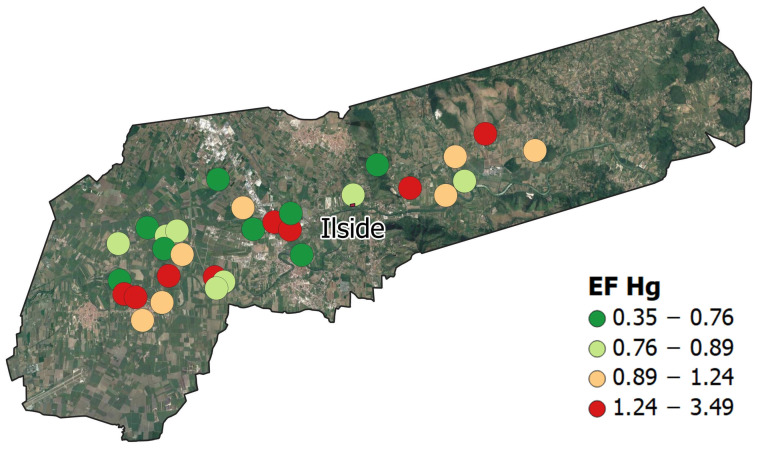
Ilside site. Dot maps of the Hg EFs distribution.

A discrete (dot) distribution map of Hg EFs was generated using the QGIS 3.22 software (Figure 4). Four different colours were assigned to symbols, in maps based on the quantile classification method. The spatial distribution pattern of the values shows that the highest enrichments are equally distributed toward the NE and SW, along the wind direction recorded at the site during the fire event (Figure 1b).

### 3.2. Vesuvian Area

**Figure 5:**
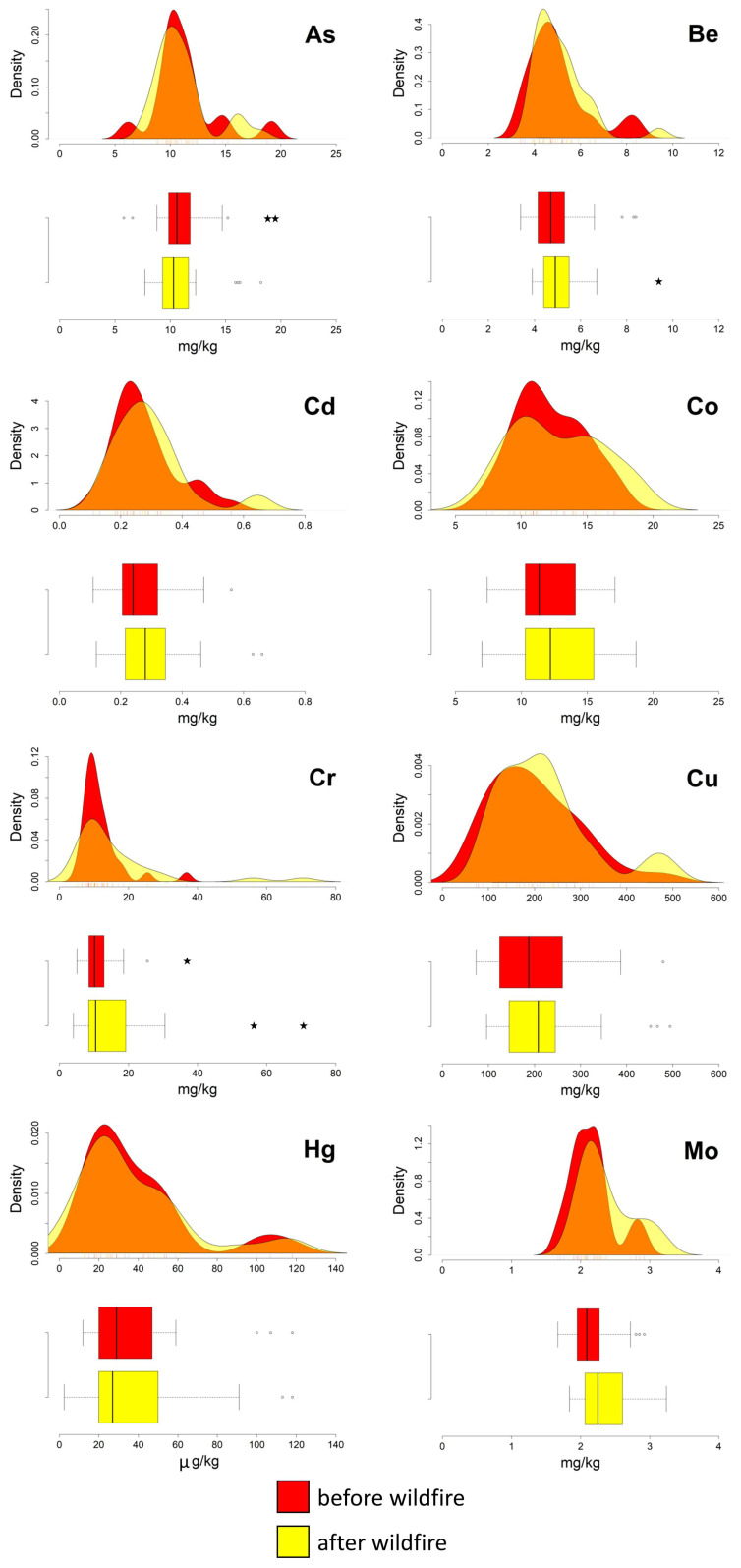
Vesuvian area. Boxplots and density curves of selected PTEs (As, Be, Cd, Co, Cr, Cu, Hg, and Mo) in the soil before and after the fire event. Circles and pentagrams represent outliers and extreme values, respectively.

**Figure 6:**
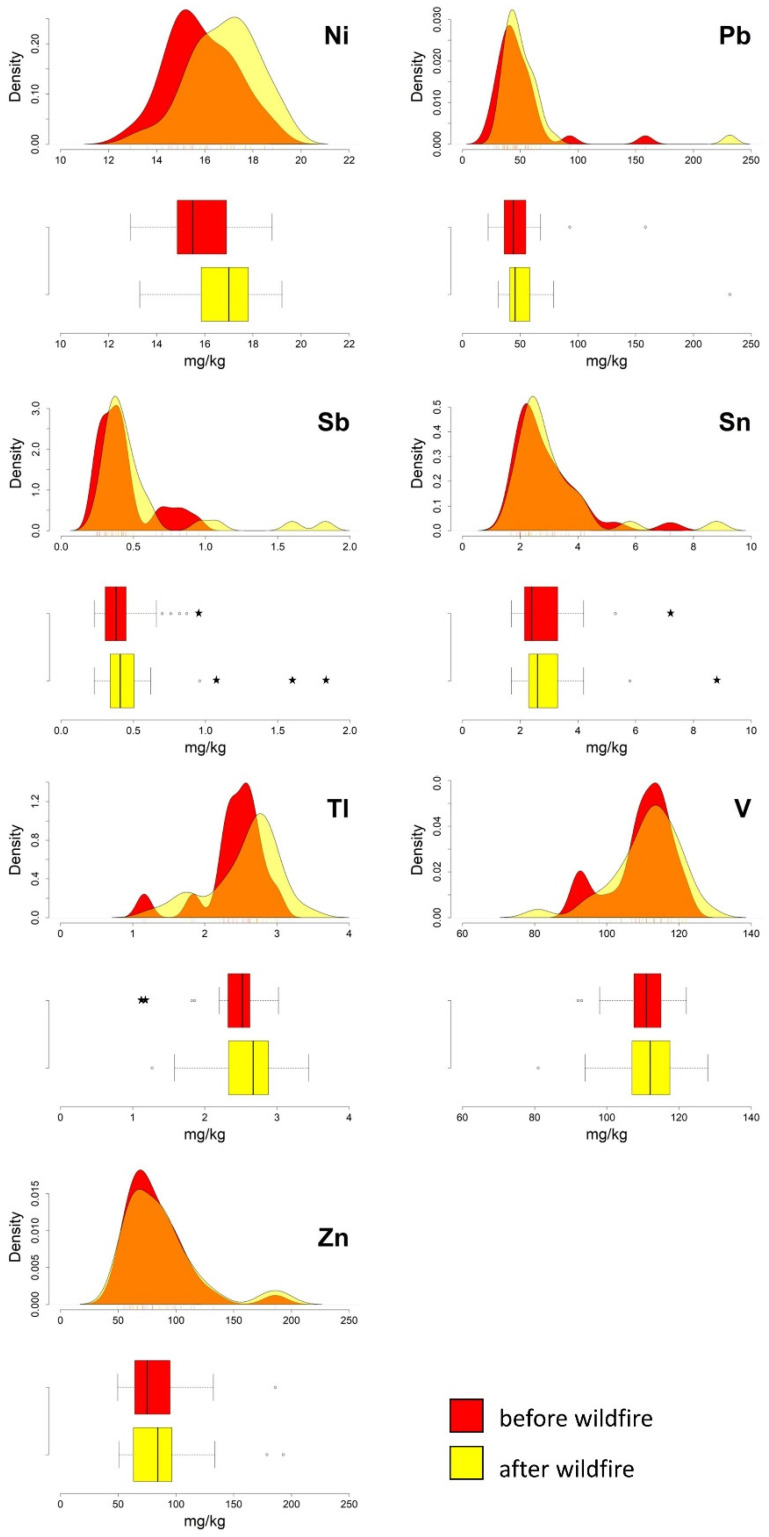
Vesuvian area. Boxplots and density curves of selected PTEs (Ni, Pb, Sb, Sn, Tl, V and Zn) in the soil before and after the fire event. Circles and pentagrams represent outliers and extreme values, respectively.

The boxplots in Figure 5 and Figure 6 show that most of the PTEs, excluding As, Cu, and Sn, are characterised by a general widening of the interquartile range, with a shift of the third quartile (75th percentile) toward higher values in the post-event data. Following the ash deposition, the median values of Mo, Ni, and Tl became even higher than the upper quartile values determined in the pre-event data (Table 2). Furthermore, Cr, and subordinately Pb, also show significant changes in their standard deviation values when comparing the two datasets (from 6.1 to 14.3 and from 24.7 to 34.7, respectively).

The graphical comparison of the density curves generated for all the PTEs in pre- and post-event data show slight changes in their distribution, except for Cu, similar to the findings obtained for the Ilside site. In the case of Cu, a change from a unimodal to bimodal distribution can be deduced from a mere visual analysis of the relative curves.

Some outliers were detected in the pre- and post-fire datasets for As, Be, Cr, Sb, and Sn. It is worth noting that Sb passed from one outlier to three outliers, and Cr from one outlier to two outliers. However, the results of the Wilcoxon test (Table 2) show that the distribution of Sb in both datasets is not affected by statistically significant changes, suggesting that outliers could depend on local processes, requiring further investigation, rather than ash deposition.

Enrichment factors (EFs) were also estimated (Table S3) for the soil surrounding the volcano. Again, iron was chosen as a normalising element for both datasets, due to it having the lowest CV in the pre- (6.44%) and post-event (7.12 %) data. 

Considering that the changes that occurred to almost all variables were significant based on the Wilcoxon test outcomes (Table 2), an RPCA was performed for a comprehensive analysis of the overall variability of the enrichments and a better understanding of the role of every single variable within the process (Table 3). 

**Figure 7:**
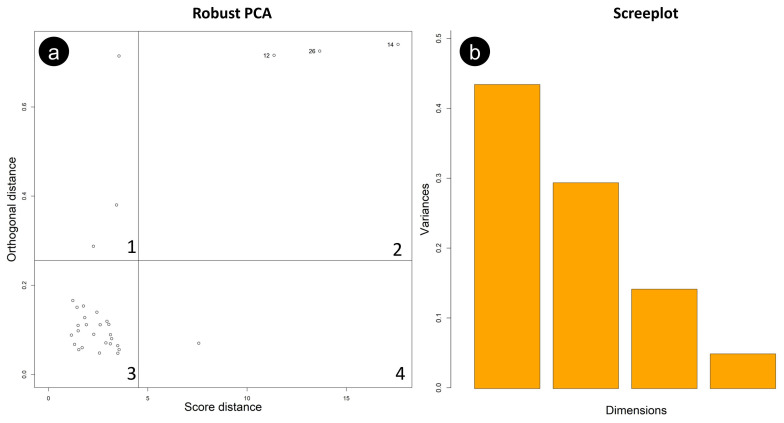
(**a**) RPCA diagnostic plot; (**b**) scree plot.

An RPCA diagnostic plot (Figure 7a), also known as an “outlier map”, allowed the separation (within the dataset) regular observations from outliers, whose influence should be limited to obtain a robust assessment of the contribution of individual variables. The score distance of each observation is reported on the horizontal axis of the plot, while the vertical axis reports the orthogonal distance of each observation to the RPCA subspace. To classify the observations, two cut-off lines, with an exceedance probability of 2.5%, were drawn [26]. Three orthogonal outliers in sector one and one good leverage point in sector four were identified and kept with regular observations to perform RPCA. Three bad leverage points (labelled with numbers 12, 14, and 26) were identified in sector two and excluded from the analysis, since they are at a sizeable orthogonal distance from the RPCA subspace and an excessive score distance from typical, expected values.

A four-component RPCA solution was chosen. Based on the variance explained by each of the components extracted by the RPCA (Table 3, Figure 7b), three PCs (accounting together for 94.6 % of the total variance) were considered here. Specifically, PC1 accounts for 47.2%, PC2 for 32%, and PC3 for 15.4%, respectively. 

**Figure 8:**
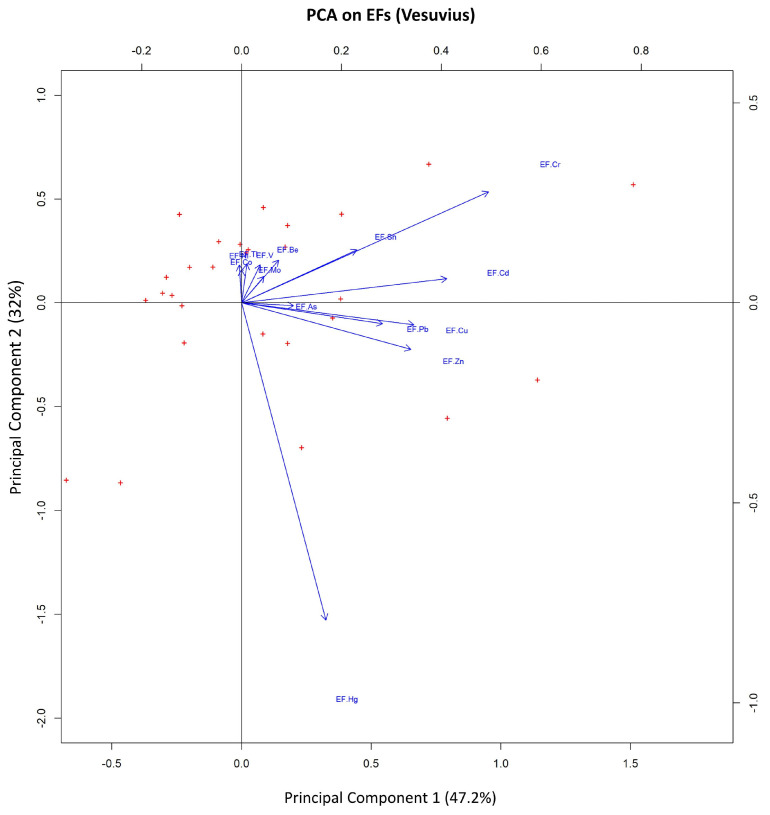
Biplots of PC1 vs. PC2. Observations (samples) are reported as red “+”, and the magnitude of the vectors (blue arrow) shows the strength of their contribution to each PC. Vectors pointing in similar directions indicate positively correlated variables, vectors pointing in opposite directions indicate negatively correlated variables, and vectors at approximately right angles indicate low or no correlation.

Using an indicative cut-off value of about 0.32 for loadings [29,30], EFs of Cr, Cd, Cu, and Zn have been considered representative of PC1, EFs of Hg and, subordinately, Cr have been selected for PC2 and, finally, EFs of Sn, Be, and V have been associated with PC3 (Table 3). The representative elements chosen for PC1 are all positively correlated among themselves. Conversely, the second component (PC2) shows a strong dependence on the Hg EF’s variability, negatively correlated with the Cr EF (Figure 8). The third component (PC3) shows significant loadings for the EFs of Sn, Be, and V (Table 3).

**Figure 9:**
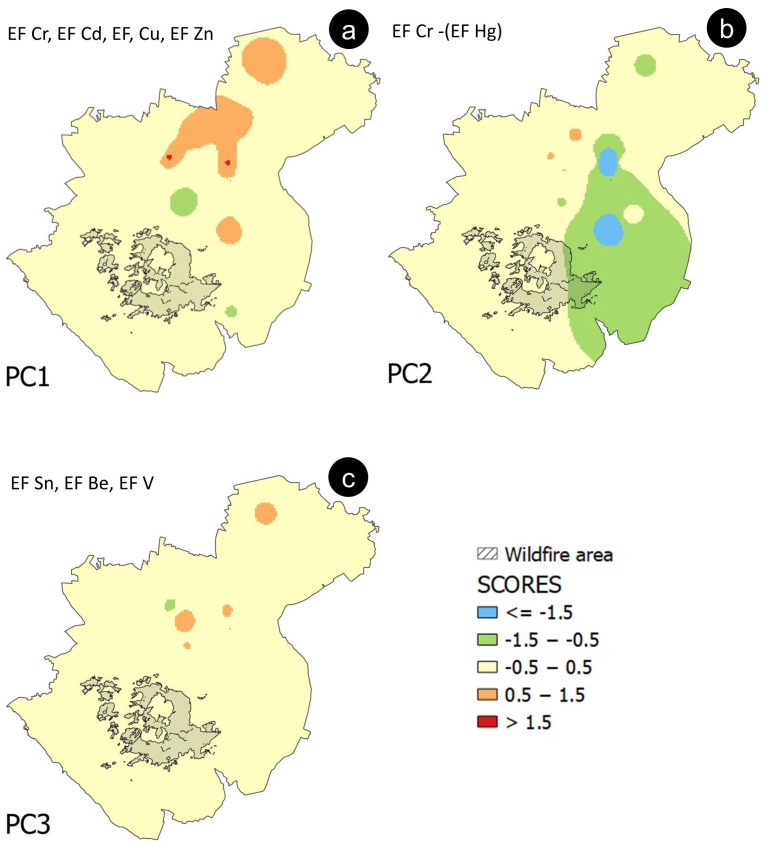
Interpolated maps of scores assigned to observations (samples) related to (**a**) PC1, (**b**) PC2, and (**c**) PC3.

The interpolated map of PC1 scores (Figure 9a) shows values higher than 0.5, mainly in correspondence with an area located NE of the volcanic complex, suggesting that this could be the area of the principal fallout of the wildfire ash proceeding from biomass (orchards, vineyards, woods).

As regards the distribution pattern of the negative scores of PC2 (Figure 9b), it is interesting to note that, although the direction of elongation of their shape is roughly consistent with the direction of the positive scores of PC1, they are located in the SE sector of the volcanic complex.

The distribution of the PC3 scores (Figure 9c) refers to an association of elements (i.e., Sn, Be, and V), which is not characterised by high individual EF values (i.e., their maximum never exceeds 2) (Table S3). 

## 4. Discussion

Although several studies [31,32,33] affirm that Hg may derive from natural sources, the markedly elevated values of Hg EFs in the soils surrounding the Ilside site, suggest that some anthropogenic processes, such as the disposal of special waste (including e-waste) and recycling activities, can cause the release and the enrichment of this element [34,35,36,37,38] in the environment, and even more so when combustion occurs [39]. The changes to the statistical distributions of Sn and Zn (from unimodal to bimodal) and the high EF values found for some PTEs (Table S2), also support the possibility that e-waste could have been involved in the combustion process at the Ilside site [40,41]. Tin and lead enrichments in the soil can be associated with burning microelectronic components, whose circuits have historically been soldered using Sn–Pb alloys [42]. Furthermore, since the Restriction of Hazardous Substances Directive (RoHS), adopted in 2006 by the European Union (EU), brought about the phase-out of Sn–Pb solders in favour of lead-free ones [43] (made of varying percentages of different PTEs), the enrichments found for Ag, Bi, Cu, Ni, and Zn could be partly explained, once again, by the presence of e-waste in the burned material.

It is also possible to relate the enrichments of Cd, Cu, Ni, and Zn with the combustion of CTR displays, plastics, electronic components (i.e., printed circuit boards), and rechargeable batteries [44,45,46] at the Ilside site, as well, Cr and Co enrichments could be associated with the incineration of LCD monitors and chipped PC-boards [40,45].

Regarding the Vesuvian area, the RPCA results show that for PC1, the EFs association can be related to a large amount of biomass involved in wildfires, as reported by Demirbas [11]. In that study, the content of PTEs in the ash produced from several types of wood and plants was investigated, and high levels of Cr (and Cd) were associated with the burning of both beech and oak wood (which are widely present on the Vesuvian slopes). The presence of Cu and Zn in the PC1 association can be potentially related to the agricultural fields involved in the fire. Orchards and vineyards are extensively cultivated on the Vesuvian slopes (mainly on the NE side), and Cu and Zn are representative elements of agricultural activities, due to their use in producing fungicides and fertilisers, respectively [47,48]. In addition, Cr can also be linked to agriculture, since it is a typical constituent of phosphate fertilisers [49], which local farmers also use. The NE elongated shape of the distribution pattern of the highest PC1 scores (Figure 9a) fits well with the available information relating to the direction of the winds during the fire, although the influence of morphology of the Mt. Somma-Vesuvius caldera edge on the dispersion pathway of the ash plume cannot be excluded.

Assuming the fire event is the common triggering source for the processes identified by the RPCA, a difference could be made based on the burned material. By observing the distribution pattern of the scores of PC2, which Hg strongly influences, it is possible to hypothesise that the fire at Mt. Somma-Vesuvius probably involved waste. This hypothesis is supported by the information provided by Manzo et al. [50], which reports a considerable number of quarries (converted into landfill sites) and landfill sites, especially in correspondence with the eastern slopes of the volcanic complex.

The score values of PC3 (Table 2) show significant loadings for the EFs of some elements (i.e., Sn, Be, and V) usually associated with volcanic soils [51]. They mainly vary within the range of values closest to zero (i.e., between −0.5 and 0.5) (Figure 9c), defining a “baseline” contribution of their association to the whole territory.

The obtained results for both the study areas highlight that the soil affected by the fallout of ash is enriched in Hg; regardless of the assessment of possible sources, this evidence could be relevant to improving the interpretation of the available local epidemiological data [52]. Mercury is, in fact, ranked third by the US Government Agency for Toxic Substances and Disease Registry among the most toxic elements [53]. It is one of the causative agents for various disorders [54], and exposure to high levels of this metal harms the brain, heart, kidneys, lungs, and immune system [55]. The deposition of ash enriched in Hg on agricultural soils may affect the growth of vegetables intended for human and animal consumption, and can favour elemental bioavailability. Since many plants tend to accumulate Hg in their roots and the shoots, this may result in elevated intake of the element for humans and animals that feed on those plants, possibly adversely affecting their health [56].

## 5. Conclusions

The analysis of the enrichments found in the soil surrounding the Ilside site allowed us to link the presence of some PTEs to the burning of waste; it also offered an interpretation key for the results related to the fire that occurred at the Mt. Somma-Vesuvius slopes, where both forest biomasses and waste were burned.

In fact, although one of the areas affected by the fire is completely included in the Vesuvius National Park, and is supposedly uncontaminated, multivariate statistics allowed the discrimination among two different categories of burned material and even to roughly individuate the disposal locations of the waste.

Apart from the specific results obtained concerning the examined case studies, the methods applied can be seen as a viable option to determine the compositional characteristics of materials burned during a fire event, even with the prospect of improving the eventual assessment process of the related environmental hazards. Considering that several physical variables can also influence the fate of the ash generated by fires, applying the proposed technique could not always be successful, especially when the chemical signal generated by the fire is not strong enough. Further studies, considering the isotopic ratio signature of burned materials, could represent the next step of the current research.

## Figures and Tables

**Table 1:** Ilside site. Descriptive statistics of the PTEs in the soil before and after the fire event. *p*-value ranges: *p* > 0.05 not significant; *p* < 0.05 significant; *p* < 0.01 very significant; and *p* < 0.001 highly significant.

Elements	UM.	Pre-Event						Post-Event						Wilcoxon Test (*p*-Value)
		Mean	Median	SD	Min.	Max.	CV %	Mean	Median	SD	Min.	Max.	CV %	
As	mg/kg	8.7	7.5	4.7	3.7	19.8	54.4	9.3	8.2	4.1	5.4	20.2	43.8	>0.05
Be	mg/kg	3.4	3.1	2.7	0.6	10.1	81.7	3.6	3.1	2.8	1.0	12.1	77.5	>0.05
Cd	mg/kg	0.3	0.3	0.1	0.2	0.6	32.1	0.3	0.3	0.1	0.1	0.4	32.0	>0.05
Co	mg/kg	11.9	11.4	2.6	7.4	17.1	22.2	12.3	12.2	3.4	7.0	18.7	27.8	>0.05
Cr	mg/kg	23.5	24.3	9.7	11.4	42.5	41.4	24.9	26.5	11.2	10.7	44.8	45.1	>0.05
Cu	mg/kg	33.3	33.8	17.0	17.3	102.7	50.9	34.4	37.0	16.7	14.7	99.2	48.7	>0.05
Hg	µg/kg	37.2	32.0	46.5	17.0	246.0	125.0	39.4	38.5	95.3	12.0	544.0	242.1	<0.001
Mo	mg/kg	0.6	0.6	0.2	0.3	1.2	39.2	0.7	0.6	0.2	0.4	1.3	31.6	>0.05
Ni	mg/kg	22.9	26.5	10.2	9.6	44.0	44.7	23.8	27.4	11.7	9.6	42.7	49.0	>0.05
Pb	mg/kg	33.9	33.0	20.1	15.0	103.5	59.2	33.0	29.9	13.5	21.2	69.8	41.0	>0.05
Sb	mg/kg	0.4	0.4	0.2	0.2	1.1	57.7	0.4	0.3	0.2	0.2	1.1	51.3	>0.05
Sn	mg/kg	2.2	1.9	1.4	0.9	7.5	65.4	2.2	1.9	1.1	1.1	4.8	50.7	>0.05
Tl	mg/kg	0.9	0.8	0.7	0.4	2.3	76.9	0.9	0.8	0.6	0.5	2.5	64.8	>0.05
V	mg/kg	51.1	50.0	15.4	34.0	83.0	30.1	50.6	49.5	11.4	36.0	76.0	22.5	>0.05
Zn	mg/kg	76.5	78.3	19.7	45.8	113.7	25.8	80.1	79.9	19.6	50.6	126.8	24.5	>0.05

**Table 2:** Vesuvian area. Descriptive statistics of the PTEs in the soil before and after the fire event. *p*-values ranges: *p* > 0.05 not significant; *p* < 0.05 significant; *p* < 0.01 very significant; and *p* < 0.001 highly significant.

Elements	U.M.	Pre-Event						Post-Event						
		Mean	Median	SD	Min	Max	CV %	Mean	Median	SD	Min	Max	CV %	Wilcoxon Test (*p*-Value)
As	mg/kg	10.9	10.6	2.9	5.8	19.5	26.1	10.8	10.3	2.5	7.7	18.2	23.3	<0.001
Be	mg/kg	4.9	4.7	1.3	3.4	8.4	27.2	5.0	4.9	1.1	3.9	9.4	22.7	<0.001
Cd	mg/kg	0.3	0.2	0.1	0.1	0.6	40.1	0.3	0.3	0.1	0.1	0.7	44.5	<0.01
Co	mg/kg	14.5	14.7	0.9	12.3	16.5	6.5	15.6	15.7	1.2	12.3	17.8	7.7	<0.001
Cr	mg/kg	10.9	10.2	6.1	5.1	36.8	56.3	12.9	10.5	14.3	4.1	70.5	110.9	<0.001
Cu	mg/kg	179.6	187.4	97.2	73.3	479.0	54.1	201.8	208.3	105.0	95.9	494.1	52.1	<0.01
Hg	µg/kg	31.7	29.0	27.1	12.0	118.0	85.4	27.8	27.0	28.4	2.5	118.0	102.2	<0.01
Mo	mg/kg	2.1	2.1	0.3	1.7	2.9	14.9	2.3	2.3	0.4	1.8	3.2	16.3	<0.001
Ni	mg/kg	15.8	15.5	1.4	12.9	18.8	8.9	16.8	17.0	1.4	13.3	19.2	8.4	>0.05
Pb	mg/kg	45.2	44.2	24.7	22.4	158.4	54.6	50.1	45.6	34.7	31.1	231.5	69.2	<0.001
Sb	mg/kg	0.4	0.4	0.2	0.2	1.0	49.0	0.5	0.4	0.4	0.2	1.8	78.4	>0.05
Sn	mg/kg	2.7	2.4	1.2	1.7	7.2	42.3	2.8	2.6	1.4	1.7	8.8	49.2	<0.001
Tl	mg/kg	2.4	2.5	0.4	1.1	3.0	18.1	2.5	2.7	0.5	1.3	3.4	19.8	<0.001
V	mg/kg	109.2	111.0	8.5	92.0	122.0	7.8	110.6	112.0	9.5	81.0	128.0	8.6	<0.001
Zn	mg/kg	79.7	75.0	27.5	49.5	186.0	34.5	82.4	84.3	33.4	50.5	192.9	40.5	<0.001

**Table 3:** Summary of RPCA performed on the EFs (Vesuvian area). Loading for every single variable is reported for the selected components.

Variables	Component Loadings			
	PC1	PC2	PC3	PC4
EF Cu	**0.37906**	−0.06128	−0.24662	−0.27492
EF Pb	0.30986	−0.05849	0.03943	−0.43021
EF Zn	**0.37260**	−0.13029	0.09138	0.03309
EF Ni	−0.00464	0.10624	0.23379	−0.09956
EF Co	−0.00019	0.09208	0.26406	−0.21240
EF As	0.11451	−0.00806	0.24031	−0.25473
EF Cd	**0.45135**	0.06777	−0.08473	0.06963
EF V	0.04136	0.10698	**0.33261**	−0.29346
EF Cr	**0.54309**	**0.31085**	−0.28046	0.14055
EF Tl	0.01194	0.10951	0.28508	−0.32642
EF Hg	0.18554	**−0.88855**	0.17617	0.09239
EF Sn	0.25353	0.14844	**0.45925**	0.54156
EF Be	0.08172	0.11956	**0.43785**	0.22637
EF Mo	0.04936	0.07418	0.18902	−0.21907
Standard deviation	0.6560	0.5398	0.3753	0.2218
Proportion of variance	0.4720	0.3196	0.1545	0.0540
Percentage of explained variance	47.2%	32.0%	15.4%	5.4%
Cumulative proportion of variance	0.4720	0.7916	0.9460	1

The bold was used to highlight the representative EFs for each Principal Component.

## Data Availability

The datasets used in the current study are available from the funding institution (IZSM), that is responsible for them, upon reasonable request.

## Supplementary Materials

The following supporting information can be downloaded at: https://www.mdpi.com/article/10.3390/ijerph20054513/s1, Table S1. PTEs and related Health hazard. Table S2. Univariate statistics of the Enrichment Factors (EFs) determined for the soil samples collected in the surrounding of the Ilside site. Table S3. Univariate statistics of the Enrichment Factors (EFs) determined for the soil samples collected in the Vesuvian area.
