# Supplementary material for: Wildfires as a Source of Potentially Toxic Elements (PTEs) in Soil: A Case Study from Campania Region (Italy)
Source: Int J Environ Res Public Health. 2023 Mar 3;20(5):4513. doi: 10.3390/ijerph20054513 (PMC10001887; doi:10.3390/ijerph20054513)
Supplement: Supplementary file 1 [file ijerph-20-04513-s001.zip › ijerph-2235324-supplementary.pdf]

Supplementary Material

Table S1. PTEs and related Health hazard.

Element	Health effects
Antimony (Sb)	Pneumoconiosis, cardiac disorders, stomach pain, diarrhoea, stomach ulcers, skin cancers, lung cancer, etc.
Arsenic (As)	Vomiting, abdominal pain, fever, diarrhoea, weakness, muscle aches, headaches, drowsiness, seizures, encephalopathy, polyneuritis, coma, seizures, oedema, hyperkeratosis, hyper pigmentation, exfoliative dermatitis, haemolysis, anaemia, hypertension, blood vessel damage, cardiomyopathy, ventricular arrhythmias, diabetes mellitus, pulmonary disorders, cancer, renal tubular acidosis, intestinal haemorrhage, etc.
Beryllium (Be)	lung disease (acute and chronic), fatigue, weight loss, chest pain, coughing, dyspnea, interstitial pulmonary fibrosis
Cadmium (Cd)	Fatigue, nausea, vomiting, teeth yellowing, osteomalacia, osteoporosis, abdominal cramps, diarrhoea, emphysema, pulmonary oedema, dyspnoea, tachycardia, chronic anaemia, cyanosis, anosmia, proteinuria, hypercalciuria, renal tubular dysfunction, cancer, etc.
Chromium (Cr)	Diarrhoea, vomiting, acidosis, skin diseases, cancer of lungs and respiratory tract, neural defects, foetal deaths, kidney diseases, and ulcers and lesions on the kidneys, liver, and myocardium
Cobalt (Co)	Nausea, tinnitus, vomiting, nerve damage, respiratory problems, anorexia, goiter, cancer, and cardiovascular and renal damages
Copper (Cu)	Diabetes; cardiac, renal, neurological, immunological, and hepatic problems; pregnancy-related complications; Wilson's disease; etc.
Lead (Pb)	Lethargy, allergies, mental retardation, depression, anxiety, dysarthria, pallor, abdominal pain, anorexia, anaemia, ataxia, paralysis, reproductive disorders, encephalopathy, hypertension, insomnia, peripheral, papilledema, neuropathy, renal disorders, hyperproteinemia, cancer, etc.
Mercury (Hg)	Nausea, vomiting, depression, lethargy, gingivitis, dyspnoea, pulmonary oedema, lung damage, pneumonia, memory loss, cerebellar ataxia, choreoathetosis, polyneuropathy, seizures, dysarthria, mad hatter syndrome, hypertension, blindness, acrodynia, erythema, hyperesthesia, acute renal failure, cancer, etc.
Molybdenum (Mo)	Severe gastrointestinal irritation, diarrhea, coma and death from cardiac failure are the symptoms of acute molybdenosis (only animal studies)
Nickel (Ni)	Lung cancer, pulmonary fibrosis, disorders related to cardiovascular, gastrointestinal, haematological, musculoskeletal, and hepatic systems; etc.
Thallium (Tl)	Somnolence; nausea; hematemesis; alopecia; polyneuropathy; cranial nerve palsies; cerebellar ataxia; optic atrophy; retrobulbar neuritis; ophthalmoplegia; renal and cardiac failure; coma; etc.
Tin (Sn)	stomachache, anemia, liver and kidney problems, nervous system function, death.
Vanadium (V)	Neurological, reproductive, gastrointestinal, immunological, and lymphoreticular disorders, cancer, etc.
Zinc (Zn)	Liver, reproductive, neurological, and developmental dysfunctions, etc.

NORD (National Organization for Rare Diseases) (2019) Rare disease database. Heavy Metal Poisoning. https://rarediseases.org/rare-diseases/heavy-metal-poisoning/. Accessed February 2023

Table S2. Univariate statistics of the Enrichment Factors (EFs) determined for the soil samples collected in the surrounding of the Ilside site.

**Enrichment Factor**	**Mean**	**Median**	**Min**	**Max**
EF Mo	0.968217	0.977110	0.434451	1.373147
EF Cu	1.060023	1.012055	0.351698	1.981881
EF Pb	1.150634	1.029926	0.481488	4.419513
EF Zn	0.994855	0.955980	0.727317	1.724233
EF Ni	1.066976	1.009039	0.303996	2.653187
EF Co	1.018711	1.011307	0.533160	1.830350
EF As	0.985403	0.951726	0.411972	1.655629
EF Cd	1.246235	1.118370	0.471854	2.813589
EF Sb	1.084327	1.022842	0.390663	1.952809
EF V	1.037075	1.022935	0.880301	1.301994
EF Cr	1.040532	0.997020	0.330227	2.960399
EF Tl	1.048693	0.963461	0.351296	2.511331
EF Hg	1.099757	0.890654	0.346668	3.485063
EF Sn	1.106063	1.011792	0.379741	2.485964
EF Be	1.186221	0.858025	0.165864	5.410596

Table S3. Univariate statistics of the Enrichment Factors (EFs) determined for the soil samples collected in the Vesuvian area.

**Enrichment Factor**	**Mean**	**Median**	**Min**	**Max**
EF Cu	0.953123	0.860905	0.181444	2.906472
EF Pb	0.910055	0.86728	0.525009	1.46785
EF Zn	0.962654	0.977057	0.457717	1.666331
EF Ni	0.921176	0.913216	0.640284	1.338828
EF Co	0.913417	0.887417	0.612232	1.490708
EF As	1.004972	0.958274	0.662338	2.054702
EF Cd	0.962438	1.028049	0.181704	1.313783
EF Sb	0.933512	0.861857	0.208932	2.447409
EF V	0.97042	0.933628	0.596054	1.828924
EF Cr	0.943314	0.911748	0.127429	2.422761
EF Tl	0.932772	0.908029	0.590313	1.619048
EF Hg	1.35715	0.963434	0.535119	6.683959
EF Sn	0.971911	0.901235	0.485558	1.519196
EF Be	0.966262	0.973327	0.537485	1.422559
EF Mo	0.894455	0.875848	0.682204	1.451863
